# Intra-uterine Asphyxia, with Report of Three Cases

**Published:** 1893-09

**Authors:** Geo. F. Hulbert

**Affiliations:** Professor of Principles and Practice of Medicine and Clinical Gynecology, Marion Sims College of Medicine, St. Louis, Mo.


Buffalo Medical and Surgical Journal

VOL. XXXIII. SEPTEMBER, 1893. NO. 2.

### Original Communications.

### INTRA-UTERINE ASPHYXIA, WITH REPORT OF THREE CASES.

BY GEO. F. HULBERT, M. D.,

Professor of Principles and Practice of Medicine and Clinical Gynecology, Marion Sims College of Medicine, St. Louis, Mo.

TO THOSE who have had the experience of believing and declaring that the child in utero was living and that everything was all right, and looked forward to a happy termination of the parturition then in hand, may possibly from time to time realize the chagrin and sense of defeat, when, upon the birth, the child has been found to be practically dead; yet not dead, for the heart still beats with a strong but slow pulsation. Every effort at resuscitation is resorted to; you are cognizant of the fact of air penetrating and distending the alveolar spaces, evidenced by the increased vigor and rapidity of the heart pulsations; and yet, in spite of that, after long continued effort, we gradually see slip through our hands the remaining spark of life, and the child is, in fact, dead.

It having been my good fortune, or misfortune, to have three experiences of this character occurring in my obstetrical work, I deemed it possibly of interest, and hope it will be of some value and satisfaction, to those who have had, or may have, experience of this kind, to peruse these cases and draw their deductions therefrom.

A similarity of circumstances and conditions appertaining to the child, as well as to the mother and the character of the labors, and the fact that the normal limits were not apparently encroached upon in any respect, as well as the fact of the peculiar condition, briefly detailed above, has emboldened me to call attention to the conditions, and to place on record these cases, not that they are especially new, or not but that many others may have had the same or like experience, yet the fact remains that the literature does not abound with information, or anything specially calling attention to the classes of cases under consideration.

The first case was delivered in a perfectly natural way, by natural forces; the two subsequent ones were made instrumental deliveries advisedly, simply for the reason that I felt the possibility of a repetition of the first. In all of them I am certain and positive that I heard the fetal heart not longer than one-half hour before delivery was accomplished; heard it distinctly but somewhat weakened in its force. At the time of delivery, in all, the appearance presented by the children as they came through the vulva was one of extreme pallor, with slight, if any, evidence of cyanosis, save a deepened tinge of the lips, with absolute muscular relaxation; there being apparently no response on the part of the muscles to any irritation that might be made. The heart was pulsating at the rate of from forty to fifty per minute. The usual methods of artificial respiration were resorted to in all, to the extent of introducing the catheter into the trachea, and thereby insuring a passage of air into the bronchial tree and alveolar spaces. That the air did penetrate, was further evinced by the slight crepitus, easily heard during compression of the chest in the expiratory part of the respiratory act. Artificial respiration was maintained until the heart ceased beating; until it ceased to respond to the stimulus presented by the areation of the blood by the artificial respiration. In none of them were there any external evidences of pathological conditions, development having been well accomplished. The umbilical cords were not without the normal limits, and it was only when the placenta was reached that there were any conditions present that might possibly account for the peculiar condition of the children. Here in all three of the cases were there found blood-clots, occupying the placental surface in over half of its area. These clots were well formed, intimately attached to the placental tissue, smooth upon the uterine side. There was nothing indicating the fact that the clot had been torn from the uterine surface, but rather that placental separation had taken place and the clot had formed and become adherent to the placental tissue. So far as the mothers were concerned, they were in average good health—the first, primipara; the last two, multipara. The character of labor was not over twelve hours' duration, and presented nothing attracting special attention, outside of the fact that the first was one of those cases which might be termed "piston rod" form of delivery, in that the head was forced down to the perineum at each pain, and upon the disappearance of the pain promptly receded to the brim. This was frequently repeated, so much so that I seriously contemplated applying the forceps, and regret that I did not, in order to enable the head to catch beneath the symphysis. The character of the pains was not sustained and vigorous in any of them, but rather more short and inefficient. In none of these cases was chloroform used; and in only one—the first —a rectal injection of twenty grains of chloral given during the first stage. The element of compression upon the head was not at all excessive and would not have attracted attention.

In the last two cases there was no pulsation appreciable in the cord; the first case I have no record of this fact. The children were separated from the mothers immediately, and in the first a small amount of blood was permitted to flow through the severed end of the cord. Unfortunately, in none of them did I obtain a post-mortem, and, as before stated, the only anatomical lesion that I can testify to is the presence of the blood-clot occupying an extensive part of the area of the placental surface. There was only a moderate amount of the amniotic fluid following the birth of each child. In the first two cases, dilatation was well advanced before the membranes ruptured; in the last case, rupture occurred at the beginning of dilatation. The points for consideration in the above are:

(*a*) The presence of the fetal heart pulsations so soon before delivery.

(*b*) The normal character of the labor.

(*c*) The extreme pallor and relaxation of the child at birth.

(*d*) The presence of the cardiac pulsation after delivery.

(*e*) The absence of any attempt upon the part of the child to perform the respiratory act.

(*f*) The response upon the part of the heart to the benefits of accomplished artificial respiration.

(*g*) The anatomical conditions presented at the placenta.

The presence of the condition "*a*" is, and justly can be, considered one of the evidences of safety, as far as the child is concerned. That the conditions presented in the subsequent developments in these three cases could be accomplished in one-half hour, is a possibility, but hardly a probability. The practical conclusion to draw at this point is, that there was going on, or had been going on, a gradual separation of the placenta from the uterine surface and the development of this clot without any outward manifestations in the way of an accidental or concealed hemorrhage. There were none of the symptoms presented by the mothers which are usually considered to indicate this state of affairs. The only variation from the normal was, as stated before, the evident weakening of the force of the fetal heart action. I testify to this as a fact appreciated by me in the repeated examinations made from the beginning to the time when this loss of strength became apparent. The conclusion we draw here is, that the possibility of placental separation may be determined by the evidences of weakening fetal heart vigor developing during the course of delivery, even though there may be no external evidences presented in the way of hemorrhages, or subjective symptoms in the way of sudden or severe localized pain. It simply emphasizes the necessity for judicially repeated examinations in regard to not only the presence of the fetal heart, but the character of its action and comparisons from one examination to another.

Under "*b*" we have classified those patients as presenting what may be termed normal deliveries, and yet there was an element of incompetency and inefficiency in the character of the uterine contractions, in that unnecessary repeated efforts were made to attain the usual and necessary results. It is possible that in this fact of inefficiency in the character of the pains lies an explanation for the premature placental separation, for it is well known that inefficiency in uterine contractions only too frequently means localized unrythmical areas of contraction, and during the course of the delivery these more or less localized areas in contracting have so disturbed the placental attachments as to break and separate them; for it is easy to conceive that the area of contraction may possibly occupy one particular part of the placental surface, whereas the area of relaxation may occupy another and at the same moment, and that necessarily there must be more or less irregular traction brought to bear upon the tissue, insuring adhesion of the placenta to the uterine wall.

Certain it is, that as far as the normal concept is concerned, the element of compression or long continued compression cannot be accepted as contributing to the conditions presented by the child. The conclusion that we draw here is the positive influence of irregular areas of contraction, being a contributory cause to premature placental separation; and taken in conjunction with the conclusion drawn from the conditions presented in "*a*," may be of considerable importance and value in the future conduct of the delivery.

The conditions presented under "*c*," to my mind, simply exhibit the final stage of asphyxiation, in that the vigor and vitality of the child has become so profoundly affected that the nerve centers failed to respond to the usual stimulus, all sufficient in the healthy living child at birth ; and we have simply left enough vitality for the time being to perpetuate and continue the heart action. I conceive that so far as the nervous system is concerned, it is a general systemic condition, and its manifestation is only too apparent in the extreme and persistent pallor and profound and permanent relaxation of the voluntary muscular system. I do not see where we can account for this state of affairs on any other hypothesis than that it was asphyxia, gradually brought about by the placental separation. This, possibly of itself, would not be sufficient to accomplish such a profound degree of asphyxia as to be the cause of the conditions presented, but with this lesion present, with this much support taken away from the child in utero, and the presentation of the subsequent compression which, under ordinary circumstances, would be natural and normal, and this continued only within normal limits, is sufficient to consume the lowered vitality that must necessarily have been present, evidenced in the change of the character of the fetal heart pulsations, and would accomplish the conditions presented at birth.

The conditions presented under "*b*" clearly establish the fact that somewhere there was resident and remaining, vitality sufficient to carry on cardiac action, and let it be understood that the character of this cardiac pulsation was what would be considered good and sufficient for the propulsion of the blood from the systemic channels. To those who have witnessed this state of affairs and examined the child in this condition, whose heart was acting at the rate usually observed—of from forty to fifty per minute—steady and regular, it can be understood that my purpose at resuscitation would be strengthened, and that I should feel a certain degree of hope and look forward to a happy termination, providing successful efforts were made to introduce air into the lungs. This hope was readily increased by responses, positive and heartily appreciated after each séance of artificial respiration, for almost immediately did the cardiac pulsations increase in rapidity, and finally attain a normal rate and vigor, yet at no time in any of these children was there the slightest response toward a relief of the conditions mentioned under "*e*," namely, an absolute want of respiratory effort on the part of the child. The only positive conclusion or deduction to draw from these two conditions being present was, that, as far as the respiratory centers were concerned, irritability had been absolutely annihilated, and the nerve tissue had reached that state where the possibility of functional activity was gone. Certainly, with the conditions presented by "*d*" and "*f*," there was somewhere remaining, in the organism of the child, sufficient vital energy, or nerve centers sufficiently active to respond to the invigorating influences presented by the artificial respiration. Exclude, as we must, the respiratory centers from any part in this evidence of remaining vitality, we are narrowed down to those situated within the cardiac muscle itself; for, bearing in mind the fact of the dual function of the pneumogastric, it is hardly rational to conclude that the invigorating influence could be carried so far distant as it would necessarily have to be in order to influence the cardiac centers situated at the origin of this nerve. For, if the nutritive changes have been so profound as to influence the respiratory centers, certainly the presumption would be that the not far distant cardiac centers, or those centers presiding over the cardiac action in the origin of the pneumogastric nerve, would remain active. Hence, the only possible explanation we can offer is, that the artificial respiration aerating the blood, which must necessarily return into the heart, thence through the aortic valves, thereby insuring fresh arterial supply to the cardiac tissue through the coronary arteries, and in this way maintaining the cardiac activity. That this could be long continued with the assumed conditions at the more distant centers, is not at all probable, and the time must sooner or later come when, even here, the nutritive changes must take place which established the molecular death of the child.

Somatically, so far as the systematic nerves and muscular systems were concerned, the children were dead; molecularly speaking, they were living—only at the heart. It has occurred to me, and probably will to those who peruse this report, that the proper explanation of the peculiar condition of these children is, that there was a fault or lesion in development, and that, regardless of any other influences, these children would probably have presented the same conditions, and the results would have been the same, whether the placental attachments were maintained or not. This is simply jumping at conclusions, and is, scientifically speaking, at variance with the testimony at hand. True it is that we have none of the evidences that might possibly be revealed in a post-mortem and microscopical examination of the parts concerned in the conditions presented, and it is only the want of this that prevents me from speaking authoritatively in regard to the proper interpretation to place upon the phenomena presented. Notwithstanding in our search for the truth, scientifically speaking, we can only be guided by the evidences and testimony at hand, at the same time freely confessing what we lack in order to establish beyond peradventure that we have arrived at the truth. Hence, our interpretation and conclusion is, that the conditions presented under "*d*," "*e*," and "*f*," are to be accounted for as explained above. Finally, under "*g*," do we present the only single tangible evidence as the cause to the effects attained. How long a fetus in utero can live, and how much of the area of the placental tissue is absolutely necessary for this life, can justly be a subject for differences of opinion. Mathematically speaking, if one-half of the area of the placental attachment becomes inoperative, one-half of the vitality of the child up to the normal standard should be sacrificed; two-thirds in area of placental separation would reduce the possibility of a normally sustained vitality two-thirds. In these cases all of two-thirds of the placental area was involved; hence, the child probably only had one-third of its capacity to exist present during some part of the process of parturition.

The moment at which this separation is begun is undetermined. The only point that can be given in evidence is, that only up to within one-half hour of delivery were there positive evidences of some disturbances having taken place, influencing the functional vigor of the fetal heart. After that time up to the point of delivery was probably brought to bear the final impressions which were sufficient to culminate in the conditions observed. Artificial respiration was continued for from one-half to three-quarters of an hour, when the heart ceased beating. The practical conclusions and lessons to draw from the foregoing, or possibly the query to propound: "Is it possible to appreciate the conditions necessary to establish intra-uterine asphyxia, and the best and probable means to resort to in order to save the child when these conditions become operative, or reasonably inferred?" The only symptoms heretofore in my experience which I have felt presented the possibility of this condition, were those described to accidental or concealed hemorrhage, namely, more or less persistent loss of blood through the vagina, or sudden and severe localized pain at that part of the uterine globe at which the placental souffle presents its greatest intensity, with more or less faintness and prostration manifest upon the part of the mother. Evidently, from the above, we must acknowledge that intra-uterine asphyxiation is a possibility without evidence of this character, and the only symptoms which may suggest the presence of this danger may be a gradual developing cardiac weakening in the fetal heart sounds, associated with incompetency and irregularity in the uterine contractions, and to the character of the sounds of the fetal heart must we largely turn for evidence. Not only must there be a disturbance in the vigor of the cardiac sound, but also in the rate, and it rationally must present the same characteristics as we would expect in asphyxia ex utero, namely, a decrease gradually and steadily in the pulse rate. Hence, where we find during the course of parturition a gradual weakening fetal heart, with a decrease in the rate associated with incompetency and inefficiency of uterine contractions, although there may be absent any other visible signs of placental separation, we are justified in assuming such condition to exist. Certain it is, if we have associated with this condition of the fetal heart the usual symptoms of placental separation, the determination of the possibility, or the presence of intra-uterine asphyxiation, need not be a difficult problem; for, if the objective evidences were present, such as hemorrhage, sudden localized pain, faintness or prostration, etc., and accompanying this we found, synchronously, a weakening in the fetal cardiac sounds, accompanied with a gradual decrease in the rate, there need be little hesitation in determining that there has been sufficient placental separation to materially influence the vitality of the child. There is one other point which was noted in the symptomatology, but which I am not sufficiently satisfied with to offer any more than a suggestion toward future attention and observation, and that is change or modification in the placental souffle, whether this sound is produced in the sinuses, situated in the uterine tissue proper, or whether it is produced in that part of the maternal circulation which dips down and comes in contact with the placental tufts related to the fetal circulation, may possibly be an open question, but the evidences at hand clearly show that as soon as placental separation is accomplished, the placental souffle disappears.

Now, this may be due to the fact that part of the maternal circulation, more properly within the placental tissue, is entirely separated; the uterine part having become condensed and contracted, and more or less occupied by blood-clots, would present difficulties in determining at which of these sites the souffle was generated. Necessarily, where there would be a placental separation, and a want of a more or less continued hemorrhage from the separated surfaces, we would probably have the same conditions accomplished, not only in the placental part of the maternal circulation, but in a large degree also in the uterine part, in that the blood channels in the placental portion would be separated in the same manner as would occur at delivery, thereby being cut off entirely, the blood ceasing to circulate through them; whereas, in the uterine part, there would occur the same tendency towards thrombosis, and from the fact that condensation in area did not occur on account of the distension of the uterine walls remaining, this clot would necessarily be more extensive and must necessarily penetrate more or less deeply into all the sinuses, which had become inoperative on account of the separation, so that rationally we should infer that there ought to be some modification in intensity, at least in the placental souffle. In the second case, it seemed to me as though I appreciated a modification of this character, but from the other fact that the physics of the condition were fully recognized, and from the fact that no record on this point is made in the first and third cases, I am slow to say positively that any modification existed in this particular phenomena. I simply present the physics as they appeared to me, and call attention to the possibility, and trust it may serve to elucidate the fact of the modification in the intensity of the placental souffle, if such does exist. It is to be hoped that others who may have had experiences of this character, or who may in the future meet these conditions, will consider the explanation of intra-uterine asphyxia of sufficient importance to make a careful and accurate report of all cases that may come under their observations. We simply in the foregoing desire to call attention to the conditions, and, as far as we are able, present testimony so far obtained, and give reasons "for the faith that is in us."

---

The investigations of Drs. Abbott and McCormick, of the Johns Hopkins University, shows that a solution containing seven per cent. of acetic acid is more effective as a germicide than bichloride of mercury.—*The Woman's Medical Journal.*

